# Corticosteroid related changes in body mass index in children and adolescents with rheumatic diseases

**DOI:** 10.1186/1546-0096-10-S1-A11

**Published:** 2012-07-13

**Authors:** Natalie Shiff, Rollin Brant, David A Cabral, Jaime Guzman, Peter B Dent, Janet E Ellsworth, Kristin M Houghton, Adam Huber, Roman Jurencak, Bianca A Lang, Maggie Larche, Claire MA LeBlanc, Paivi M Miettunen, Kiem G Oen, Johannes Roth, Claire Saint-Cyr, Rosie Scuccimarri, Leanne M Ward

**Affiliations:** 1 Dalhousie University, Halifax, NS, Canada; 2 McGill University, Montreal, QC, Canada; 3 McMaster University, Hamilton, ON, Canada; 4 National Pediatric Bone Health Working Group, Ottawa, ON, Canada; 5 Université de Montréal, Montreal, QC, Canada; 6 University of Alberta, Edmonton, AB, Canada; 7 University of British Columbia, Vancouver, BC, Canada; 8 University of Calgary, Calgary, AB, Canada; 9 University of Manitoba, Winnipeg, MB, Canada; 10 University of Ottawa, Ottawa, ON, Canada; 11 University of Saskatchewan, Saskatoon, SK, Canada

## Purpose

Corticosteroids (CS) are commonly used for the treatment of children with rheumatic diseases (RD) at presentation or relapse; the dose is reduced or discontinued with disease improvement. The aim of this study was to examine the dose-related effect of CS on the timing of peak body mass index (BMI) in children with RD and the degree of restitution to pre-CS BMI.

## Methods

We used data from the Steroid Associated Osteoporosis in the Pediatric Population (STOPP) Canadian Incidence Study for patients >age 2 years with RD from enrolment to 18 months after CS initiation. We grouped patients according to clinically meaningful CS starting doses (combining total IV and oral CS dose in the first 2 weeks and calculated as mg of prednisone-equivalent per kg of body weight per day). CS starting doses were defined as high (≥ 1.00 mg/kg/d), moderate (0.20 to 0.99 mg/kg/d) and low (<0.20 mg/kg/d to a maximum of 7.50 mg/d). We developed a mixed effects growth curve model as a non-parametric estimate of average BMI z-score trajectory, estimated using cubic splines, a type of robust polynomial regression.

## Results

**Figure 1:**
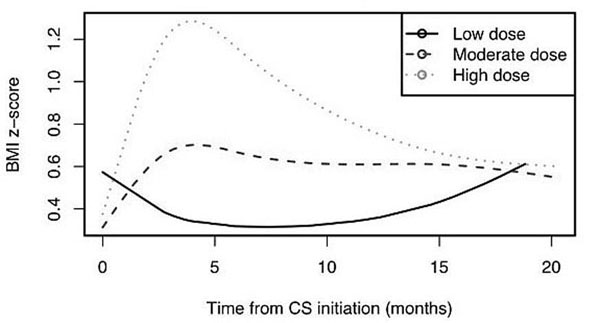
BMI z-score trajectories for children with RD according to CD starting dose

Data was available for 114 of 136 subjects (65.4% female). Median study-entry age was 10 years (inter-quartile range 6-14 years). Diagnoses were: juvenile idiopathic arthritis (38.2%), systemic lupus erythematosus (15.4%), juvenile dermatomyositis (22.1%), and other RD (24.3%). CS starting dose was high in 57.2%, moderate in 39.8% and low in 3%. BMI peaked at about 4 months after CS initiation (Figure 1). The BMI z-score curves for the moderate and high CS groups were significantly different (p<0.001). The low dose group had too few subjects for analysis. Overall, 49.1% (95% CI, 39.7%, 58.6%) of subjects failed to return to within +0.25 SD of their baseline z-scores at 18 months. A similar percentage of patients in the moderate and high CS starting dose groups failed to return to within +0.25 SD of their baseline z-scores at 18 months (45.2% vs 51.1%, p=0.8). Contrary to our hypothesis, higher baseline BMI did not reduce the likelihood of returning to baseline BMI z-scores.

## Conclusion

In children with RD treated with moderate to high doses of CS, BMI increases and peaks at 4 months independent of CS starting dose. High CS starting doses produce higher peaks, but do not change the general shape of the BMI trajectory after CS initiation compared to moderate CS starting doses. Approximately half of all patients started on CS for RD fail to return to within +0.25 SD of their baseline BMI 18 months later. Funding: CIHR, UBC Clinician Investigator, Frederick Banting and Charles Best Canada Graduate Scholarships (CIHR).

## Disclosure

Natalie Shiff: None; Rollin Brant: None; David A. Cabral: None; Jaime Guzman: None; Peter B. Dent: Roche , 6; Janet E. Ellsworth: None; Kristin M. Houghton: None; Adam Huber: None; Roman Jurencak: None; Bianca A. Lang: None; Maggie Larche: None; Claire M.A. LeBlanc: None; Paivi M. Miettunen: None; Kiem G. Oen: None; Johannes Roth: None; Claire Saint-Cyr: None; Rosie Scuccimarri: None; Leanne M. Ward: None; the Canadian STOPP Consortium: None.

